# Supplementary material for: Gingival Tissue MiRNA Expression Profiling and an Analysis of Periodontitis-Specific Circulating MiRNAs
Source: Int J Mol Sci. 2023 Jul 26;24(15):11983. doi: 10.3390/ijms241511983 (PMC10418511; doi:10.3390/ijms241511983)
Supplement: Supplementary file 1 [file ijms-24-11983-s001.zip › ijms-2363017-supplementary.pdf]

Table S1. Comparison of anthropometric and sociodemographic characteristics between participants groups.

Characteristics	PD+ (*N*=144)	PD− (*N*=86)	*p*-value	Total (*N*=230)
Sex			**0.012**	
Female	116 (80.6)	80 (93.0)		196 (85.2)
Male	28 (19.4)	6 (7.0)		34 (14.8)
Age (years)	53.88 ± 10.39	47.59 ± 13.99	**<0.001**	51.53 ± 12.22
Tobacco usage			**0.011**	
Never	77(53.5)	60 (69.8)		137 (59.6)
Former	40 (27.8)	21 (24.4)		61 (26.5)
Current smoker	27 (18.8)	5 (5.8)		32 (13.9)
Alcohol consumption			0.806	
Never	42 (29.2)	28 (32.6)		70 (30.4)
Once per week	22 (15.3)	14 (16.3)		36 (15.7)
Once per month	80 (55.6)	44 (51.2)		124 (53.9)
Education			0.058	
Secondary	26 (18.1)	6 (7.0)		32 (13.9)
Higher non-university	34 (23.6)	21 (24.4)		55 (23.9)
University education	84 (58.3)	59 (68.6)		143 (62.2)
Annual dental prophylaxis			0.885	
≤ Once per year	98 (68.1)	57 (66.3)		155 (67.4)
≥ Two times per year	46 (31.9)	29 (33.7)		75 (32.6)
Tooth brushing frequency			0.345	
≤ Once per day	39 (27.1)	18 (20.99)		57 (24.8)
≥ Two times per day	105 (72.9)	68 (79.1)		173 (75.2)
Approximal teeth cleaning			**0.002**	
Yes	100 (69.4)	75 (87.2)		175 (76.1)
No	44 (30.6)	11 (12.8)		55 (23.9)
BMI (kg/m^2)	25.49 ± 4.04	24.47 ± 4.08	0.066	25.11 ± 4.08

BMI: body mass index; PD: periodontitis.

Values are presented as number (%) or mean ± standard deviation. Significant ***p***-values are presented in bold.

Table S2. List of all human (hsa-) mature miRNAs significantly deregulated (fold change ≥ 1.5 or ≤ –1.5; $p \leq 0.05$) in periodontitis-affected tissues as compared to healthy gingiva.

Mature miRNA	Change	Log_2 fold change	*p*-value
miR-3609	UP	21.097752	5.982961E-6
miR-4539	UP	13.911187	0.0034073854
miR-378c	UP	13.431882	2.5006192E-10
miR-4441	UP	12.385956	0.003198552
miR-30c-2-3p	UP	11.029368	5.418997E-6
miR-30a-3p	UP	10.593317	0.0017030366
miR-5008-5p	UP	10.312013	0.0026602696
miR-4697-5p	UP	10.024114	0.005105327
miR-3192-3p	UP	9.723486	0.020792214
miR-424-3p	UP	9.569782	0.0070886305
miR-4470	UP	8.668313	0.005056986
miR-3622b-5p	UP	8.42965	4.78392E-4
miR-378e	UP	8.383979	0.002259727
miR-3064-5p	UP	8.212205	0.014977334
miR-4633-5p	UP	7.852584	0.0011231073
miR-4418	UP	7.691243	0.022530572
miR-4513	UP	6.820944	0.006500478
miR-6894-5p	UP	6.8161774	0.0067733973
miR-7515	UP	6.53498	0.011592695
miR-6861-5p	UP	6.5065513	0.009267809
miR-4685-5p	UP	6.432738	0.0012611016
miR-3202	UP	6.3974605	0.004982368
miR-4690-5p	UP	6.3139095	0.013501005
miR-4436b-3p	UP	6.1638193	0.024903229
miR-4419b	UP	6.136029	0.005548597
miR-4769-5p	UP	6.0760255	0.011993192
miR-4496	UP	5.969935	0.009363752
miR-4429	UP	5.90162	0.0051523037
miR-4502	UP	5.797169	0.047689036
miR-4468	UP	5.77554	0.012844757
miR-6837-5p	UP	5.6663938	0.007101256
miR-4538	UP	5.601535	0.017772188
miR-6857-5p	UP	5.483419	0.018755458
miR-6856-5p	UP	4.9241266	0.01511683
miR-3620-5p	UP	4.8548417	0.04710426
miR-4776-5p	UP	4.622304	0.020126823
miR-195-3p	UP	4.517651	0.011706592
miR-4303	UP	4.3227415	0.027616186
miR-450a-5p	UP	4.2617965	0.010951824
miR-4746-5p	UP	4.232331	0.0366363
miR-330-3p	UP	4.2140427	0.006444162
miR-206	UP	4.1199203	0.042994898
miR-4436a	UP	4.060867	0.02800539
miR-23a-5p	UP	3.9183314	0.014982747
miR-3605-5p	UP	3.7601562	0.029584713
miR-6809-5p	UP	3.7239244	3.2156786E-5
miR-3934-5p	UP	3.630751	0.023955273
miR-500a-5p	UP	3.5253022	0.01454097
miR-6848-5p	UP	3.4622562	0.014737124
miR-575	UP	3.4531016	0.0011067445
miR-4260	UP	3.2527676	0.04791562
miR-145-5p	UP	3.1845005	0.021697244
miR-342-5p	UP	3.1619499	0.048123162
miR-298	UP	3.120741	0.029798966
miR-630	UP	3.0428808	0.009250191
miR-6860	UP	2.9854276	0.013185181
miR-4741	UP	2.973071	0.0012034714

miR-664b-3p	UP	2.9504206	0.005567918
miR-5703	UP	2.8355925	0.011031834
miR-6797-5p	UP	2.6717365	0.047991127
miR-4778-5p	UP	2.65862	7.407193E-5
miR-378f	UP	2.6352327	0.010649294
miR-4667-5p	UP	2.5545022	1.3657047E-4
miR-4449	UP	2.5493603	0.0019621078
miR-4485-5p	UP	2.5399292	0.017961767
miR-6740-5p	UP	2.445647	0.002228632
miR-125a-3p	UP	2.44189	0.022030998
miR-6867-5p	UP	2.4399276	2.5397007E-4
miR-4530	UP	2.4129372	0.00584117
miR-6833-5p	UP	2.346126	5.0820694E-5
miR-4299	UP	2.3412848	0.030506475
miR-6509-5p	UP	2.333467	0.0064273607
miR-195-5p	UP	2.3163059	0.038429923
miR-1273e	UP	2.310981	0.00876341
miR-4417	UP	2.2925766	0.011851156
miR-4673	UP	2.2872994	0.023105094
miR-6769b-5p	UP	2.255693	9.867043E-4
miR-1273g-3p	UP	2.2523165	0.026946027
miR-140-3p	UP	2.2518585	9.440837E-5
miR-155-5p	UP	2.2384453	0.01930039
miR-3917	UP	2.230352	0.008262329
miR-29c-5p	UP	2.1508615	0.006506333
miR-6779-5p	UP	2.1423898	0.0020925484
miR-126-5p	UP	2.120029	0.023130203
miR-4800-5p	UP	2.1096334	4.4997458E-4
miR-8071	UP	2.109437	0.0024428465
miR-30a-5p	UP	2.105474	0.012757925
miR-3135b	UP	2.063051	0.0022571874
miR-146a-5p	UP	2.060337	0.015934054
miR-3653-3p	UP	2.040937	0.04503131
miR-6845-5p	UP	1.9597281	0.0012857373
miR-6797-5p	UP	1.9570135	0.04799698
miR-3198	UP	1.9380133	0.017172765
miR-126-3p	UP	1.9271178	0.042272385
miR-4726-5p	UP	1.924321	0.039449226
miR-7846-3p	UP	1.9166954	7.393787E-4
miR-6869-5p	UP	1.9058439	0.038597155
miR-1587	UP	1.8904784	0.0021048759
miR-6785-5p	UP	1.8745447	0.028154764
miR-1236-5p	UP	1.8654809	1.9044965E-4
miR-5787	UP	1.8623778	0.034411028
miR-619-5p	UP	1.8036032	0.018589552
miR-6089	UP	1.7807527	0.010131884
miR-4688	UP	1.7667979	0.010726886
miR-1273f	UP	1.7483053	0.0023988842
miR-151b	UP	1.7471061	0.010215847
miR-663a	UP	1.7395145	0.026515884
miR-6880-5p	UP	1.7364151	0.016118988
miR-939-5p	UP	1.7321529	0.011508225
miR-146b-5p	UP	1.7312315	0.02566925
miR-4306	UP	1.7062432	0.04094264
miR-6727-5p	UP	1.6939839	0.022205204
miR-4505	UP	1.6833506	0.004957147
miR-454-3p	UP	1.6546508	0.005893807
miR-6757-5p	UP	1.6535947	9.5336325E-4
miR-4743-5p	UP	1.6515175	0.0036350158
miR-6088	UP	1.6448472	0.017500624

let-7i-5p	UP	1.6409252	0.025608461
miR-6800-5p	UP	1.6326962	0.041857917
miR-7150	UP	1.6209935	0.04087265
miR-151a-5p	UP	1.6191373	0.02545305
miR-3679-5p	UP	1.6182171	0.003557818
miR-7110-5p	UP	1.6021211	0.044018492
miR-6086	UP	1.5929787	2.3032364E-4
miR-6807-5p	UP	1.5885594	0.019388216
miR-652-3p	UP	1.5882461	0.0021738857
miR-185-5p	UP	1.584046	0.043001547
miR-4646-5p	UP	1.5774035	0.005415294
miR-6124	UP	1.5709971	0.0028802548
miR-550a-3-5p	UP	1.5602642	0.031311247
miR-423-5p	UP	1.5595804	0.0073272916
miR-6512-5p	UP	1.5541701	0.009410762
miR-4656	UP	1.5516189	0.015074907
miR-6819-5p	UP	1.5400215	0.005807056
miR-186-5p	UP	1.5377585	5.2593544E-4
miR-6831-5p	UP	1.5365962	0.012452283
miR-6778-5p	UP	1.5287228	0.008788148
miR-6510-5p	UP	1.5262713	0.02339382
miR-6780b-5p	UP	1.5160023	0.013042399
miR-4793-5p	UP	1.5123715	0.0029080445
miR-3065-3p	DOWN	−19.018412	3.860544E-4
miR-4310	DOWN	−7.878956	0.018763479
miR-4664-3p	DOWN	−5.686357	0.026030745
miR-203a-3p	DOWN	−5.1764946	0.033030972
miR-1246	DOWN	−3.7977161	3.0806994E-5
miR-4644	DOWN	−3.494985	0.033787355
miR-933	DOWN	−3.3012435	0.0017494974
miR-197-5p	DOWN	−3.2965791	6.982468E-4
miR-1290	DOWN	−3.0894573	0.004031998
miR-6824-3p	DOWN	−2.8438542	0.034796074
miR-210-3p	DOWN	−2.6959174	0.023188422
miR-4788	DOWN	−2.4317288	3.4581788E-4
miR-636	DOWN	−2.399055	0.03535577
miR-6848-3p	DOWN	−2.3979795	0.007723487
miR-6076	DOWN	−2.198916	0.03849026
miR-6777-3p	DOWN	−2.119105	0.01939316
miR-1908-3p	DOWN	−2.0877237	0.0078876065
miR-4271	DOWN	−2.061026	0.04601549
miR-1224-5p	DOWN	−2.0331528	0.02894065
miR-937-5p	DOWN	−1.969027	0.021730866
miR-6800-3p	DOWN	−1.9490974	0.003593747
miR-1304-3p	DOWN	−1.9389137	0.034340326
miR-1234-3p	DOWN	−1.9386121	0.024319692
miR-425-3p	DOWN	−1.8585198	0.022727754
miR-1238-3p	DOWN	−1.8552052	0.029386519
miR-191-3p	DOWN	−1.8225256	0.04069585
miR-1825	DOWN	−1.7523289	0.03864391
miR-6765-3p	DOWN	−1.7489574	0.030737506
miR-6126	DOWN	−1.7358894	0.0048215017
miR-1228-3p	DOWN	−1.7327114	0.020878114
miR-6069	DOWN	−1.7129666	0.043092586
miR-4665-3p	DOWN	−1.6860542	0.011447039
miR-4497	DOWN	−1.653489	0.028820565
miR-6127	DOWN	−1.5607718	0.02591698
miR-6165	DOWN	−1.5342814	0.035260037
miR-6756-5p	DOWN	−1.5064877	0.03753026
miR-4261	DOWN	−1.5037313	0.04282309

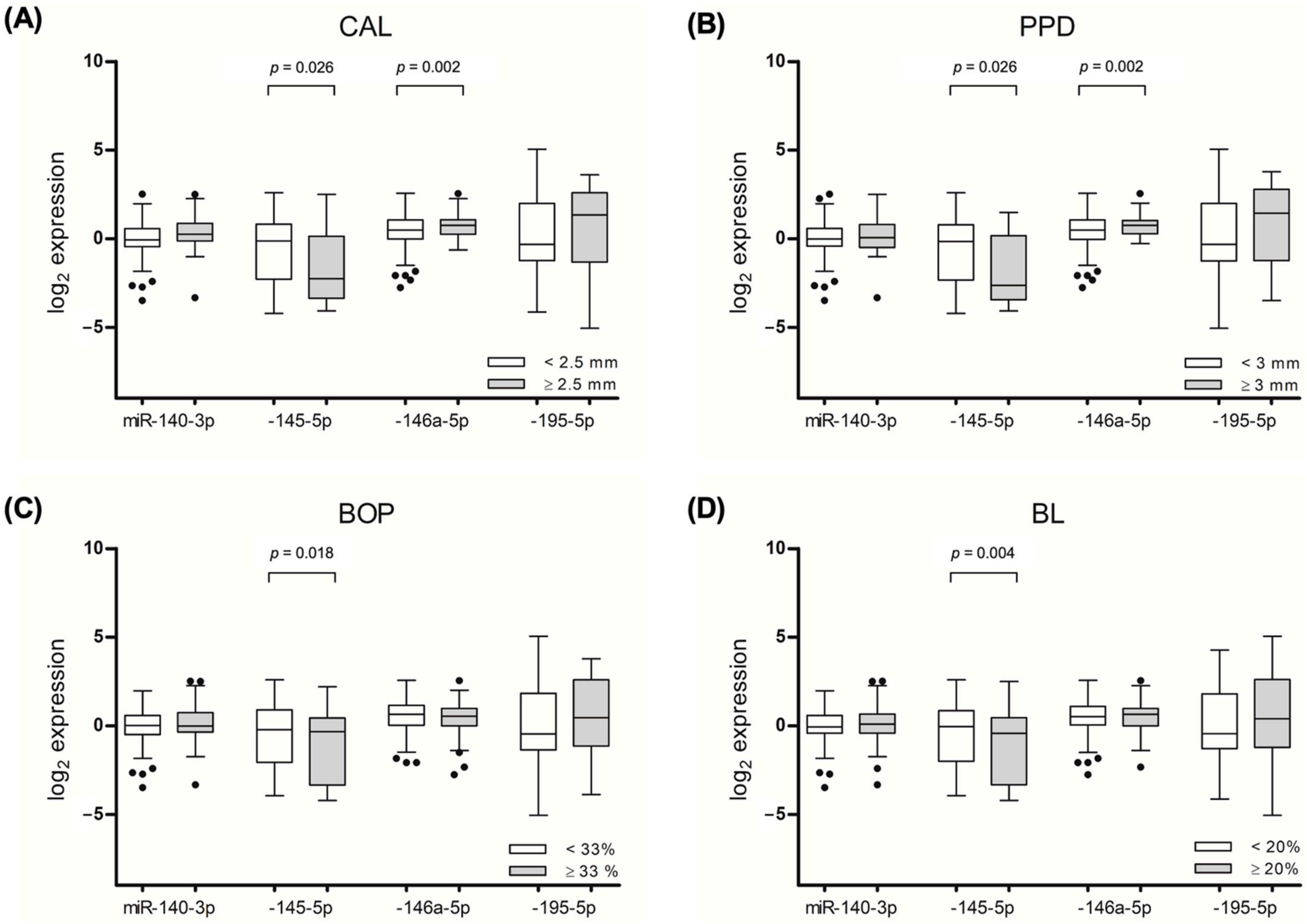

Figure S1. Comparison of blood plasma miR-140-3p, miR-145-5p, -146a-5p, and -195-5p levels with regard to cut-off points of (A) mean clinical attachment loss (CAL); (B) periodontal probing depth (PPD); (C) bleeding on probing (BOP); (D) bone loss (BL). The bands inside the boxes indicate the median, the whiskers show the data interval, and the dots represent outliers.

Table S3. Periodontal, rheumatological and tobacco usage status (*N* or mean ± SD) of study participants of genome-wide miRNA expression profiling stage.

**Periodontal status, *N***	**PD+, 8**		**PD–, 8**	
**RA status, *N***	RA+, 2	RA–, 2	RA+, 2	RA–, 2
**PD clinical parameters**				
CAL (mm)	3.25 ± 0.44	3.13 ± 0,75	1.1 ± 0.66	1.3 ± 0.70
	3.19 ± 0.57		1.2 ± 0.64	
PPD (mm)	3.23 ± 0.24	3 ± 0.12	2.13 ± 0.24	2 ± 0.27
	3.11 ± 0.21		2.06 ± 0.24	
BOP (%)	59.5 ± 11.79	53.75 ± 8.85	12.75 ± 1.5	11.75 ± 2.22
	56.63 ± 10.13		12.25 ± 1.83	
BL (proportion of root length)	36.12 ± 7.34	30.39 ± 7.26	15.4 ± 6.79	15.85 ± 5.38
	33.26 ± 7.42		15.63 ± 5.68	
Missing teeth (*N*) BOP (%)	11.5 ± 6.02	8.5 ± 3.87	4.25 ± 5.44	5 ± 4.97
	10 ± 4.96		4.63 ± 4.84	
**PD Stages**				
Stage III (*N*)	1	1	N.A.	
	2			
Stage IV (*N*)	3	3	N.A.	
	6			
**RA clinical parameters and treatment**				
DAS28-CRP (score)	4.07 ± 1.2	N.A.	3.83 ± 2.53	N.A.
RAID (score)	3.96 ± 2.65	N.A.	3.96 ± 1.0	N.A.
HAQ (score)	0.68 ± 0.5	N.A.	1.15 ± 0.16	N.A.
**Tobacco usage**				
Former (*N*)	3	3	0	0
	6		0	
Never (*N*)	1	1	4	4
	2		8	

Abbreviations: bDMARDs: biologic disease-modifying antirheumatic drugs; BL: bone loss; BOP: bleeding on probing; CAL: clinical attachment loss; DAS28-CRP: Disease Activity Score 28-joint count C reactive protein; HAQ: health assessment questionnaire; N.A.: not applicable; PD: periodontitis; PPD: periodontal probing depth; RA: rheumatoid arthritis; RAID: rheumatoid arthritis impact of disease; SD: standard deviation; sDMARDs: synthetic disease-modifying antirheumatic drugs.
